# Supplementary material for: Electrophysiological and Behavioral Evidence for Hyper- and Hyposensitivity in Rare Genetic Syndromes Associated with Autism
Source: Genes (Basel). 2022 Apr 11;13(4):671. doi: 10.3390/genes13040671 (PMC9027402; doi:10.3390/genes13040671)
Supplement: Supplementary file 1 [file genes-13-00671-s001.zip › genes-1634981-supplementary.pdf]

Supplementary Table S1. Description of the experimental ERP papers included in the review.

Reference	Experimental groups	Paradigm/task	Main results
**Fragile X syndrome**			
(Castrén и др., 2003)	5 FXS patients<br>4 control subjects	1. auditory oddball paradigm: standard tones (85% occurrence) at a frequency of 800 Hz and deviant ones at a frequency of 560 Hz<br>2. auditory gating task (four identical tones)	↑ amplitude of N1 to auditory stimuli in both tasks<br>no N1 habituation in gating task
(Rojas и др., 2001)	11 FXS patients<br>11 control subjects	1000-Hz pure tones	↑ amplitude of N100m
(Van der Molen и др., 2012b)	16 FXS patients<br>20 control subjects	auditory oddball paradigm: standard tones (90% occurrence) at a frequency of 1000 Hz and deviant ones at a frequency of 1500 Hz	↑ amplitude of N1 to standard stimuli<br>↑ amplitude of P2 to standard stimuli<br>↓ MMN amplitude<br>↓ P3a amplitude to deviant stimuli
(Ethridge и др., 2019)	38 FXS patients<br>40 control subjects	1. four 50-ms white noise bursts of 50 ms duration<br>2. chirp stimuli (white bursts modulated by sinusoid in frequency from 0 to 1000 Hz)	↑ amplitude of N1<br>↑ amplitude of P2
(Ethridge и др., 2016)	14 FXS patients<br>15 control subjects	auditory gating task (four identical tones)	↓ habituation in N1 component<br>↓ amplitude of N2
(Knoth и др., 2014)	12 FXS patients<br>12 control subjects	1. auditory modality: 50 ms broadband noise presented 150 times<br>2. visual modality: black-and-white checkerboard pattern	↑ amplitude of N1 to auditory stimuli<br>↑ amplitude of P2 to auditory stimuli<br>↑ latency of N2 to auditory stimuli<br>↑ amplitude of N70 to visual stimuli
(Côté и др., 2021)	14 FXS patients<br>55 control subjects	sequences of four vowels	↑ N1-P2 peak-to-peak value
(Van der Molen и др., 2012a)	16 FXS patients<br>22 control subjects	1. auditory modality: auditory oddball paradigm: standard tones (90% occurrence) at a frequency of 1000 Hz and deviant ones at a frequency of 1500 Hz<br>2. visual modality: blue and yellow smiley faces	↑ amplitude of N1 and N2 to both auditory and visual stimuli<br>↓ P3b amplitude
(Rigoulot и др., 2017)	17 FXS patients<br>26 control subjects	neutral faces, each repeated successively ten times	↑ N170 amplitude for the second presentation of a stimulus compared to controls
(St Clair и др., 1987)	33 FXS patients<br>83 control subjects	auditory oddball paradigm: standard tones at a frequency of 1000 Hz and deviant ones at a frequency of 1500 Hz	↑ amplitude of N1 to standard stimuli<br>↑ amplitude of P2 to standard stimuli<br>↓ P3 amplitude
**Angelman syndrome**			

(Key и др., 2018)	15 del AS patients<br>15 control subjects	Sequence of nonwords, random stimuli were repeated	Larger parietal responses within 200-500 latency were associated with better communication skills reported by caregivers
(Key & Jones, 2019)	13 del AS patients	Novel names and names of participants and their close relatives were presented	No differences in ERP components on response to novel and familiar names
(Egawa и др., 2008)	11 del AS patients<br>2 non-del AS patients<br>6 patients with epilepsy (non-AS)<br>11 control subjects	Electrical median nerve stimulation	↑ N1m and P1m latencies in del-AS compared with non-del AS patients and control subjects<br>↑ N1m amplitude in del-AS patients
(Guerrini и др., 1996)	11 AS patients	Electrical median nerve stimulation<br>Transcranial magnetic stimulation to evoke motor potentials	↑ SEPs were prolonged in 5 patients<br>↓ silent period following motor evoked potentials
**Phelan-McDermid Syndrome**			
(Grosman и др., б. д.)	5 PMS patients<br>8 ASD patients<br>9 control subjects	auditory steady-state response paradigm, ASSR (40-Hz click trains)	↓ ASSR for both PMS and ASD patients
(Neklyudova и др., 2021)	1 patient with microduplication of SHANK3 gene<br>32 control subjects	auditory steady-state response paradigm, ASSR (40-Hz click trains)	↓ ASSR
(Isenstein и др., 2018)	6 PMS patients<br>7 control subjects	1000-Hz pure tones	↓ P2 amplitude<br>↑ P2 habituation
(Reese, 2019)	37 PMS patients<br>15 control subjects	auditory gating task: broadband noise bursts in pairs	↓ P50 amplitude
(Ponson и др., 2018)	10 PMS subjects<br>no data about control group	auditory oddball paradigm: standard tones (85% occurrence) at a frequency of 1000 Hz and deviant ones at a frequency of 1100 Hz	↑ N250 latency in patients with ASD than in norm
(Brittenham, 2017)	17 PMS patients<br>24 ASD patients<br>12 control subjects	black-and-white checkerboard	↓ P60-N75 amplitude<br>↓ N75-P100 amplitude
(Siper и др., 2021)	31 PMS patients<br>79 patients with idiopathic ASD<br>45 control subjects	black-and-white checkerboard	↓ amplitude of P60-N75 deletion size was negatively correlated with P60-N75 amplitude
**Rett syndrome**			
(Sysoeva и др., 2020)	12 RS patients<br>21 control subjects	1. auditory oddball paradigm for tones: standard tones (85% occurrence) at a frequency of 1000 Hz and deviant ones at a frequency of 500 Hz<br>2. auditory oddball paradigm for phonemes: standard phonema (85% occurrence) was 'ba' and deviant one was 'da'	↑ P1 amplitude was greater for phonemes in control subjects, but not in RS patients<br>no P2 component for both tones and phonemes in RS patients<br>negative correlation between P2 amplitude and severity of symptoms

(Badr и др., 1987)	7 RS patients<br>58 control subjects	1. tone bursts<br>2. auditory oddball paradigm: standard tones (75% occurrence) at a frequency of 1000 Hz and deviant ones at a frequency of 2000 Hz<br>3. visual modality: flash stimulation	↑ N1, P2 latencies in auditory modality<br>↑ P3 latency to deviant stimuli in auditory modality<br>↑ P1 latency in visual modality
(Foxe и др., 2016)	14 RS patients<br>22 control subjects	auditory oddball paradigm: standard tones (85% occurrence) at a frequency of 503 Hz and deviant ones at a frequency of 996 Hz	↑ MMN latency
(Brima и др., 2019)	11 RS patients<br>24 control subjects	auditory oddball paradigm: standard stimuli had duration of 100 ms and deviant ones had duration of 180 ms. Interstimulus intervals were different: 450, 900 and 1800 ms	↓ MMN with interstimulus interval of 450 ms and no MMN with longer intervals
(Saunders и др., 1995)	17 RS patients<br>18 control subjects	1. visual oddball paradigm<br>2. auditory oddball paradigm: standard tones (80% occurrence) at a frequency of 440 Hz and deviant ones at a frequency of 880 Hz	no developmental changes (reduction in amplitude and latency) of P1 components to visual stimuli in RS patients<br>absence of developmental changes (reduction in amplitude and latency) of N2 components to auditory stimuli in RS patients<br>no P3 component to deviant auditory stimuli
(LeBlanc и др., 2015)	34 RS patients<br>20 control subjects	visual modality: black and white checks	↓ N1-P2 complex amplitude<br>↑ N2 latency
(Yoshikawa и др., 1991)	10 RS patients	Electrical median nerve stimulation	↑ SEP in 7 of 10 patients
(Yamanouchi и др., 1993)	9 RS patients<br>54 control subjects	visual modality: white flashed<br>somatosensory modality: electrical median nerve stimulation	↑ P30-N35 amplitude to somatosensory stimulation
**Tuberous sclerosis**			
(Côté и др., 2021)	9 TSC patients<br>55 control subjects	sequences of four vowels	↓ P1-N1 complex amplitude
(Seri и др., 1999)	14 TSC patients (7 with ASD)	auditory oddball paradigm for tones: standard tones (80% occurrence) at a frequency of 1000 Hz and deviant ones at a frequency of 1500 Hz	↓ N1 amplitude<br>↑latency subgroup with ASD<br>↑ MMN latency in subgroup with ASD
(O'Brien и др., 2020)	9 TSC patients<br>9 control subjects	auditory oddball paradigm for tones: standard tones (85% occurrence) at a frequency of 800 Hz and deviant ones at a frequency of 400 Hz<br>auditory oddball paradigm for phonemes: standard phoneme (85% occurrence) was 'a' and deviant one was 'u'	↑ P1 and N2 amplitude to vowels compared to tones in controls, but not in TSC patients<br>= MMN between control subjects and TSC, however in patients with both TSC and ASD ↓ MMN amplitude

(Jeste и др., 2013)	28 TSC patients<br>26 control subjects	presentation of pictures of a caregiver and gender- and age-matched stranger	↑ N290 latency
(Tye и др., 2015)	14 TSC patients (4 with ASD)<br>13 control subjects	Presentation of faces, either upright or inverted	↑ N170 in TSC and ASD to typical faces<br>↑ N170 to inverted faces in controls and TSC, but not in TSC with ASD
(Varcin и др., 2016)	16 infants with TSC patients<br>18 control infants	black-and-white checkerboard	no difference in amplitude of latencies of ERP components were found
**Neurofibromatosis type 1**			
(Ammendola и др., 2006)	21 NF1 patients	1. black-and-white checkerboard<br>2. electrical stimulation of posterior tibial nerve at the ankle	↑ P100 latency in visual modality<br>↑ P37 latency somatosensory modality
(Yerdelen и др., 2011)	39 NF1 patients	1. black-and-white checkerboard<br>3. electrical stimulation of the median nerve at the level of the wrist	↑ P100 latency in visual modality<br>↑ latency of somatosensory EP
(Begum-Ali и др., 2021)	25 NF1 patients (infants)<br>52 control subjects (infants)	auditory oddball paradigm for tones: standard vowels /u/ (50% occurrence) at a frequency of 500 Hz and deviant ones were /u/ at a frequency of 650 Hz (25%) of deviant vowel /i/ at a frequency of 500 Hz (25%)	↑ latency of pitch -change detection in infants with NF1
(Iannaccone и др., 2002)	16 NF1 patients<br>13 control subjects	pattern-reversal and flash stimuli	↑ P1 latency<br>absent P2 component in 3 patients
(Ribeiro и др., 2015)	16 NF1 patients<br>16 control subjects	visual Go/NoGo task	↓ P1 amplitude<br>↓ P3 amplitude<br>P3b in response to irrelevant stimuli was not reduced
(Bluschke и др., 2017)	14 NF1 patients<br>22 control subjects	Flanker task	↓ the conflict-modulation of N2 in NF1 group<br>↑ N450 amplitude of N450 component
(Pobric и др., 2021)	16 NF1 patients<br>16 control subjects	1 N-back and 2 N-back tasks	↓ P3 latency
